# Anatomy and micromorphology of *Imperata* (Poaceae, Andropogoneae) from Brazil and their taxonomic significance

**DOI:** 10.3897/phytokeys.279.207098

**Published:** 2026-09-18

**Authors:** Bianca L. S. Pinto, Cassiano A. D. Welker, Juliana Marzinek

**Affiliations:** 1 Universidade Federal de Uberlândia, Programa de Pós-graduação em Biologia Vegetal, Rua Ceará s.n., Umuarama, 38400-902, Uberlândia, Minas Gerais, Brazil Universidade Federal de Uberlândia, Programa de Pós-graduação em Biologia Vegetal Uberlândia Brazil

**Keywords:** Epidermal micromorphology, grass anatomy, leaf epidermis, leaf vascular bundles, species delimitation, taxonomy

## Abstract

Species delimitation in *Imperata* (Poaceae, Andropogoneae) is complicated by overlapping morphological variation. Here, we reassess the taxonomy of Brazilian *Imperata* and investigate leaf and culm anatomy together with leaf epidermal micromorphology to evaluate their taxonomic relevance. Field collections and herbarium specimens were examined using standard taxonomic procedures, light microscopy, and scanning electron microscopy. Three species were confirmed for Brazil (*I. brasiliensis*, *I. contracta*, and *I. tenuis*), whereas the occurrence of *I. minutiflora* in the country was not supported. We provide an identification key, updated descriptions, distribution maps, and illustrations of the confirmed species, as well as lectotype designations for *I. contracta* and *I. tenuis*. The most informative anatomical and micromorphological characters were found in the leaf blade, including the presence of dumbbell-shaped guard cells in *I. brasiliensis*, distinctive long and short epidermal cells in *I. contracta*, and the absence of cushion-hairs in *I. tenuis*. In contrast, leaf sheath and culm anatomy were comparatively more uniform. Our results support the use of comparative anatomy and micromorphology as complementary sources of evidence for the delimitation of Brazilian species of *Imperata*.

## Introduction

*Imperata* Cirillo (Poaceae, Andropogoneae) is a cosmopolitan grass genus with approximately 13 species distributed mainly in tropical and subtropical regions (Welker et al. 2020; Soreng et al. 2022). The genus includes some aggressive weeds that negatively impact agriculture and livestock production in many parts of the world, such as *I. cylindrica* (L.) Raeusch. and *I. brasiliensis* Trin. (Aronovich et al. 1973; Estep et al. 2014; Kellogg 2015). In Brazil, four species of the genus have been reported: *I. brasiliensis*, *I. contracta* (Kunth) Hitchc., *I. minutiflora* Hack., and *I. tenuis* Hack. (Flora e Funga do Brasil 2026).

*Imperata* is characterized by inflorescences densely covered with long silky trichomes and by paired spikelets arranged along a tough rachis, both pedicellate but with pedicels of unequal length. In contrast, most members of the tribe Andropogoneae have one sessile and one pedicellate spikelet at each node of the rachis, which is fragile at maturity (Flores 2001; Welker and Longhi-Wagner 2012; Kellogg 2015). Although *Imperata* was traditionally placed in the subtribe Saccharinae (Clayton and Renvoize 1986), phylogenetic studies have recovered the genus as sister to the *Apocopis–Germainia–Pogonatherum* clade, supporting its placement in Germainiinae (Welker et al. 2016, 2020; Gallaher et al. 2022; Soreng et al. 2022). This position is also consistent with morphological features shared with other members of the subtribe, including reduction of the androecium to one or two stamens and absence of lodicules (Welker et al. 2020; Gallaher et al. 2022).

Despite recent advances in the taxonomy and phylogeny of Andropogoneae, species delimitation and identification in *Imperata* remain problematic. Previous taxonomic treatments (e.g., Gabel 1982; Welker and Longhi-Wagner 2012; Peichoto 2013; Peichoto and Welker 2023) have highlighted the overlap of morphological characters among species, particularly between *I. brasiliensis* and *I. tenuis*, which have been distinguished mainly by plant height and inflorescence length. Recent molecular analyses of Neotropical *Imperata* likewise recovered limited resolution among American lineages and placed *I. brasiliensis* and *I. tenuis* close together in phylogenetic trees, with neither forming a monophyletic group (Cordobés et al. 2021). This further reinforces the need for additional sources of evidence to support species delimitation in *Imperata*.

In Poaceae, comparative leaf anatomy has long contributed to taxonomic interpretation, particularly when combined with external morphology (Metcalfe 1960; Ellis 1976, 1979). Studies using scanning electron microscopy (SEM) have likewise demonstrated the taxonomic relevance of leaf micromorphological features (e.g., Hilu 1984; Dávila and Clark 1990; Zhang et al. 2014; Khan et al. 2017). Standardized anatomical and micromorphological terminology and descriptive frameworks have improved the comparability of characters across taxa and reinforced the systematic value of features such as epidermal cells, stomatal complexes, trichomes, vascular bundle arrangement, and bundle sheaths (Metcalfe 1960; Ellis 1976, 1979). Within the tribe Andropogoneae, several studies have shown that these characters can provide useful evidence for generic and specific delimitation (e.g., Renvoize 1982; Hilu 1984; Dávila and Clark 1990; Peichoto 2003; Ahmad et al. 2010; Ullah et al. 2011; Nazir et al. 2013; Farr et al. 2024). For *Imperata*, however, anatomical and micromorphological evidence remains limited and is concentrated mainly on *I. cylindrica*. Renvoize (1982) described the leaf-blade anatomy of that species and recognized four orders of vascular bundles, one more than is usually reported for Poaceae (e.g. Metcalfe 1960; Ellis 1979). Additional studies on *I. cylindrica* emphasized the taxonomic value of epidermal and vascular characters (Ahmad et al. 2010; Ullah et al. 2011; Nazir et al. 2013). In Brazil, Falcão (1971) described the rhizome and leaf-blade anatomy of *I. brasiliensis*, but a comparative anatomical assessment across all Brazilian species is still lacking. Consequently, the potential contribution of anatomy and micromorphology to species delimitation in Brazilian *Imperata* remains insufficiently explored.

Here, we reassess the taxonomy of *Imperata* in Brazil and investigate leaf and culm anatomy, along with leaf epidermal micromorphology, to evaluate their taxonomic relevance. Specifically, we aim to: (1) re-evaluate the occurrence of *Imperata* species in Brazil and provide an identification key, updated descriptions, distribution maps, and illustrations for the confirmed taxa; and (2) describe anatomical and micromorphological characters of the Brazilian species in order to assess their contribution to species delimitation.

## Material and methods

### Plant material and resources

The taxonomic study of Brazilian *Imperata* was based on specimens collected during field expeditions and on material deposited in the following herbaria: BAA, BHCB, CEN, CEPEC, FLOR, FURB, HUCS, HUFU, IBGE, ICN, K, LE, MBM, NY, P, RB, SMDB, SP, US, and W (acronyms according to Thiers 2026). For comparative purposes, specimens of *I. minutiflora* collected in other countries were also examined.

Protologues and type material of the studied taxa were examined, together with information available in Tropicos (2026) and JSTOR Global Plants (JSTOR 2026). When necessary, lectotypifications were carried out for *Imperata* names. The specimens designated here as lectotypes conform to the protologue of the respective taxa and are the best-preserved specimens from the corresponding type collections.

Morphological descriptions, taxonomic comments, distribution maps, and illustrations were prepared for each *Imperata* species confirmed for Brazil. An identification key to the species is also provided, adapted from Welker and Longhi-Wagner (2012) and expanded to include anatomical and micromorphological characters documented in this study. Measurements and descriptions were based on characters previously recognized as taxonomically informative in the genus (Gabel 1982; Flores 2001; Scrivanti et al. 2012; Welker and Longhi-Wagner 2012; Peichoto 2013; Peichoto and Welker 2023; Flora e Funga do Brasil 2026). Habit, inflorescences, spikelets, and other diagnostic characters of the confirmed species were illustrated in ink by the botanical illustrator Klei Rodrigo Sousa. Selected specimens examined are cited in the text, with only one specimen listed per Brazilian state. A complete list of specimens examined is provided in Suppl. material 1.

Distribution maps were prepared for each species in QGIS version 3.40, based on all specimens examined in this study. When herbarium specimens lacked original geographic coordinates, approximate coordinates were assigned based on the locality descriptions provided on the specimen labels.

### Anatomy and micromorphology

Anatomical and micromorphological analyses were performed for all *Imperata* species confirmed to occur in Brazil. For each species, three herbarium specimens were selected for these studies: *I. brasiliensis* (*Carniello et al. 1002*, HUFU; *Guimarães & Falkenberg 470*, HUFU; *Klein & Bresolin 8826*, HUFU), *I. contracta* (*Valls 2667*, ICN; *Valls et al. 3291*, ICN; *Valls & Arzivenco 1384*, ICN), and *I. tenuis* (*Araújo 2392*, HUFU; *Oliveira 402*, HUFU; *Welker 219*, ICN).

For leaf anatomy, one fully developed leaf was sampled from each specimen. Four regions were analyzed: the leaf sheath and the basal, median, and apical portions of the leaf blade. Transverse sections were obtained from all regions to assess tissue composition and structural continuity along the leaf. Paradermal sections of the median portion of the leaf blade were prepared to support the identification of epidermal cell types. For standardization, anatomical descriptions were based primarily on the median portion of the leaf blade, and deviations observed in other regions were recorded when relevant.

Samples were rehydrated in 3% NaOH for 20 min following Anderson (1963), with modifications, and subsequently washed in distilled water for 36 h. The material was then dehydrated through an ascending ethanol series, infiltrated, and embedded in historesin (Leica Biosystems). After polymerization, the embedded material was sectioned on a Leica RM2235 rotary microtome to obtain transverse sections 6 µm thick and paradermal sections 4 µm thick. Sections were stained with 0.05% toluidine blue in acetate buffer, pH 4.7, as described by O’Brien et al. (1964), with modifications. Permanent slides were mounted in synthetic resin (Entellan, Merck) and examined under light microscopy. Images were captured using an Olympus BX51 microscope and digitally organized for plate preparation.

For culm anatomy, internodes were sampled and processed using the same rehydration, dehydration, and embedding procedures described above. For culm analysis, transverse sections were 4 µm thick.

Micromorphological analyses were conducted using scanning electron microscopy (SEM). Leaf samples were kept in a humid chamber for 48 h to restore flexibility, then mounted on aluminum stubs, sputter-coated with gold, and examined under a Tescan VEGA 3 LMU scanning electron microscope.

Anatomical descriptions of the leaf blade in transverse section followed Ellis (1976), whereas epidermal characters in surface view followed Ellis (1979). General anatomical terminology for Poaceae followed Metcalfe (1960).

## Results

### Taxonomic reassessment and treatment

The taxonomic reassessment of Brazilian *Imperata* confirmed the occurrence of three species in the country: *I. brasiliensis*, *I. contracta*, and *I. tenuis*. The occurrence of *I. minutiflora* in Brazil has not been confirmed. No specimens of this species from Brazil were found during field trips conducted over several years, nor in the herbaria examined or in the databases consulted. The Brazilian specimens previously identified under that name (*Chase 12033*, RB, US) in fact correspond to *I. tenuis*. No additional evidence of *I. minutiflora* in Brazil was found, and the species is therefore excluded from the Brazilian flora.

An identification key, updated descriptions, distribution maps, and illustrations are provided below for the confirmed species, along with lectotype designations for *I. contracta* and *I. tenuis*. Complete specimen lists are presented in Suppl. material 1.

### Key to the Brazilian species of *Imperata*

**Table:** 

1	Inflorescences subcontracted, (12–)24–51 cm long; basal branches divergent from the main axis. Leaf blade epidermis with long cells rectangular or pentagonal, and with short cells dumbbell-shaped	***Imperata contracta***
–	Inflorescences contracted, 4–27 cm long; basal branches appressed to the main axis. Leaf blade epidermis with long cells rectangular, and with short cells rectangular or dumbbell-shaped	**2**
2	Inflorescences 4–16(–18) cm long. Plants (21–)32–95(–120) cm tall. Leaf blade epidermis with cushion-hairs; stomata with guard cells elongated or dumbbell-shaped	***Imperata brasiliensis***
–	Inflorescences (10–)14–27 cm long. Plants (66–)81–177 cm tall. Leaf blade epidermis without cushion-hairs; stomata with guard cells elongated	***Imperata tenuis***

#### 1. Imperata brasiliensis

Taxon classificationPlantaePoalesPoaceae

Trin., Mém. Acad. Imp. Sci. St.-Pétersbourg, Sér. 6, Sci. Math. 2(3): 331. 1832.

92D65683-3CFB-5892-BFCF-62A43CE5006B

Figs 1, 2 A, B

##### Type.

Brazil • Minas Gerais: Serra da Lapa, Nov. 1823, *L. Riedel 1016* (lectotype, designated by Pinto et al. 2023: LE [LE00000639, digital image!]; isolectotype: K [K000632954!]).

**Figure 1.**
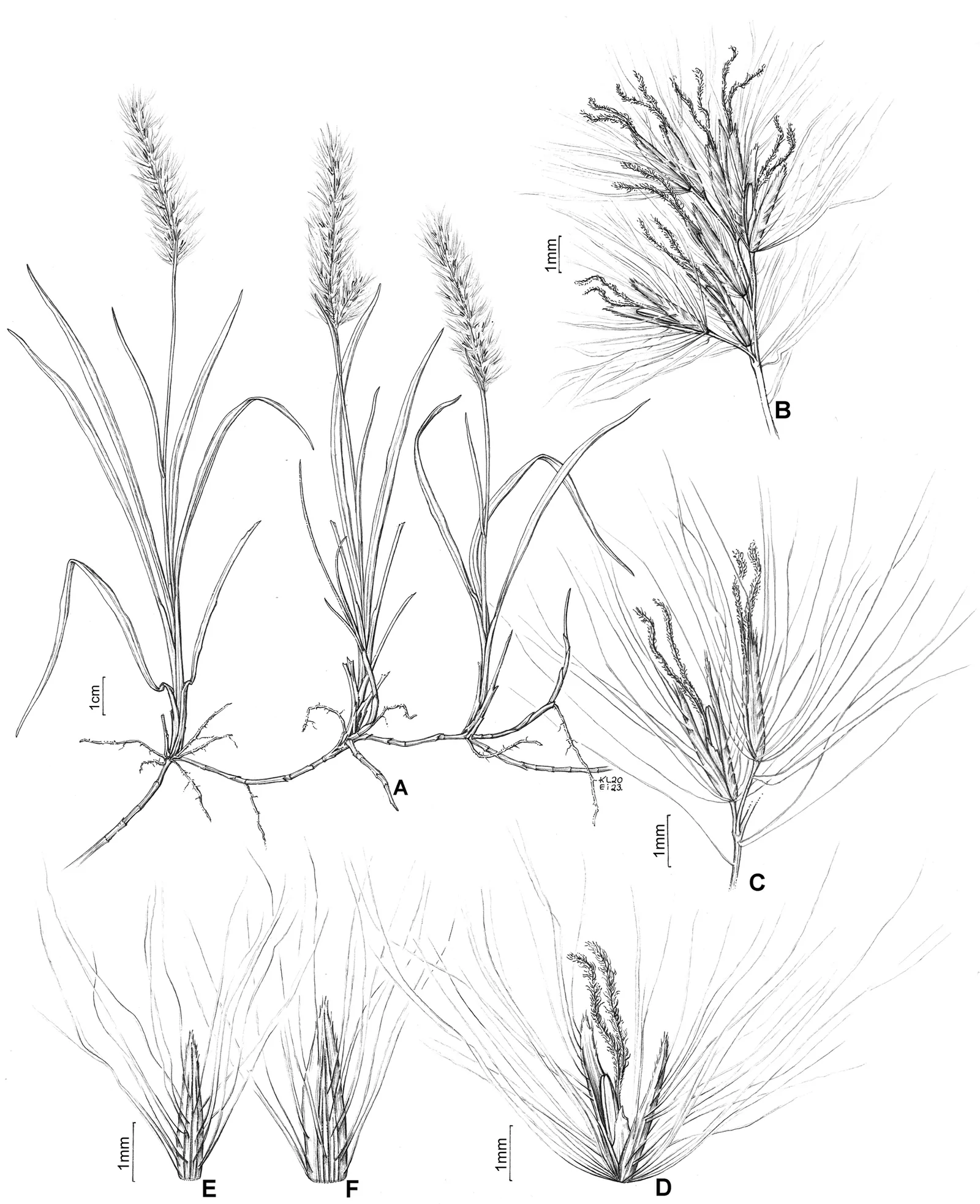
*Imperata brasiliensis*. **A**. Habit; **B**. Portion of the inflorescence; **C**. Pair of spikelets; **D**. Spikelet showing stigma and anther; **E**. Lower glume; **F**. Upper glume. Based on *Klein & Bresolin 8826* (HUFU).

**Figure 2.**
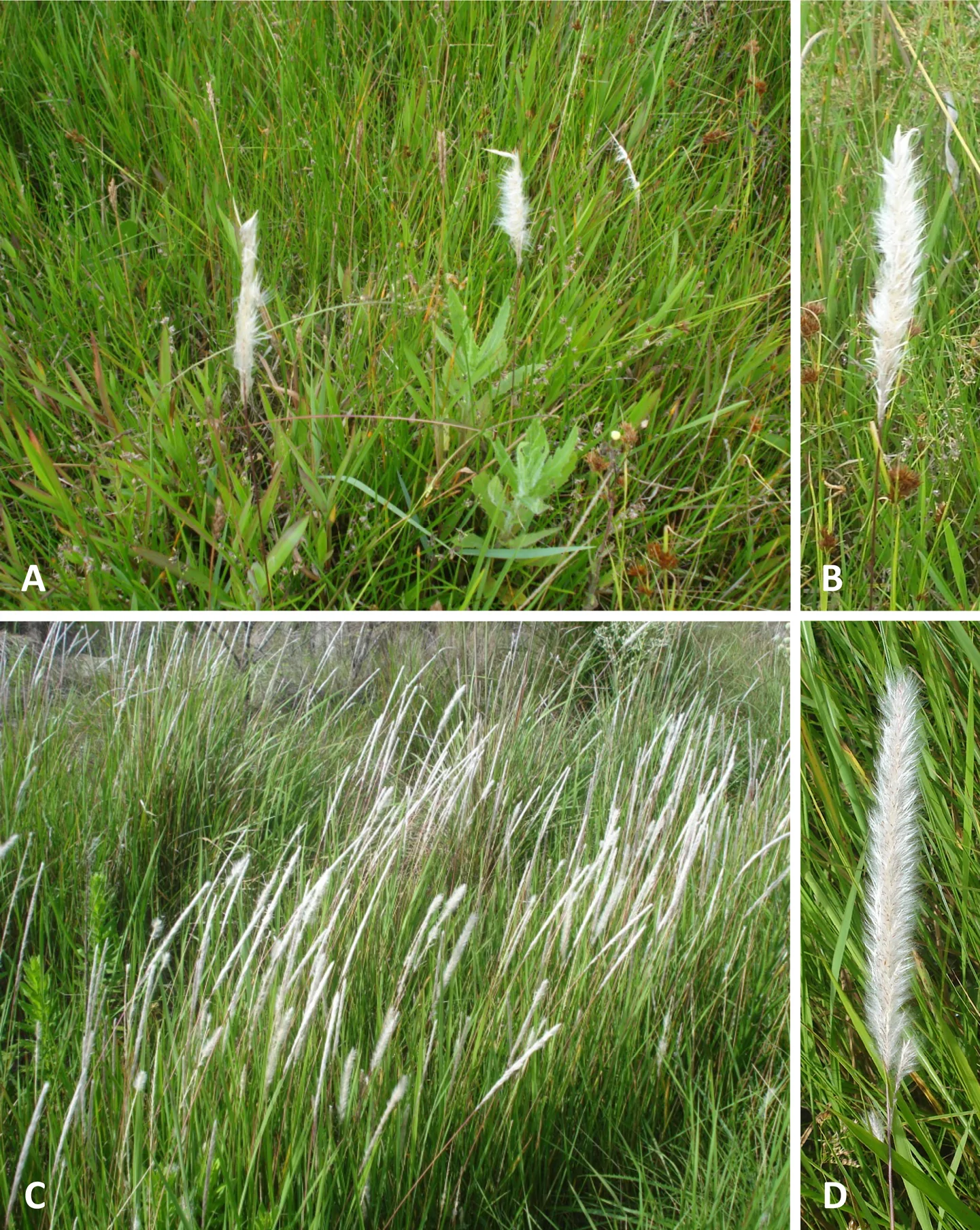
Field photographs of *Imperata* species. **A, B**. *I. brasiliensis*. **A**. Habit; **B**. Inflorescence. **C, D**. *I. tenuis*. **C**. Habit; **D**. Inflorescence. Photos by C.A.D. Welker.

##### Description.

Plants perennial, (21–)32–95(–120) cm tall. Culms erect; nodes glabrous; internodes hollow. Leaf sheaths glabrous. Ligules membranous-ciliate, 1–2.5(–3) mm long. Leaf blades (6.5–)13–49(–58) × 0.2–1.5(–2.1) cm, linear-lanceolate, rarely lanceolate, pseudopetiolate, with pseudopetiole generally not reduced to the leaf midrib; abaxial surface glabrous; adaxial surface pilose, with a higher concentration of trichomes in the basal region and tufts of trichomes near the ligule; margins with prickle-hairs. Inflorescences 4–16(–18) cm long, contracted; basal branches (0.5–)0.9–3.5(–4.5) cm long, appressed to the main axis. Rachis tough, bearing pairs of spikelets, both pedicellate, with pedicels of unequal length. Spikelets (2.1–)2.3–5.2 mm long, muticous, with long silky white trichomes; lower glume (2–)2.3–4(–4.8) mm long; upper glume (2.1–)2.8–4.5(–5.1) mm long. Lower floret sterile; lower lemma 1–3.5 mm long; lower palea absent. Upper floret bisexual; upper lemma 0.5–1.1 mm long; upper palea 0.6–1.4 mm long. Stigmas 2, vinaceous, 2–5 mm long. Stamen 1; anther (1.3–)2–3.2(–4) mm long, yellow to orange-brown. Caryopsis 0.9–2 mm long.

##### Distribution and habitat.

**Figure 3.**
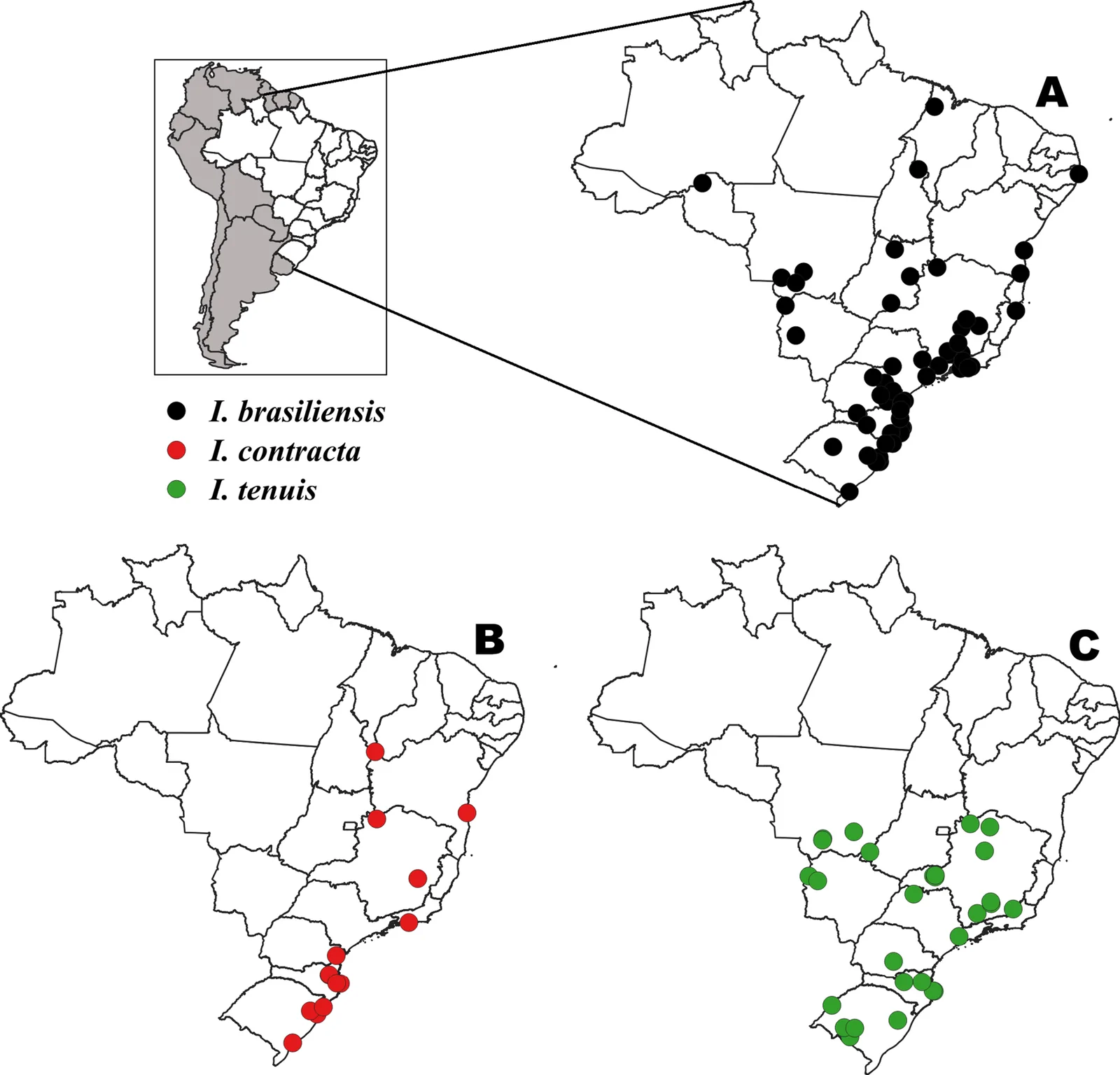
Distribution map of *Imperata* species in Brazil based on the herbarium specimens examined in this study. **A**. *I. brasiliensis*; **B**. *I. contracta*; **C**. *I. tenuis*.

Widely distributed from the United States of America to Argentina, Uruguay, and Brazil (Filgueiras 2003; Scrivanti et al. 2012). In Brazil, the species occurs throughout most of the country (Fig. 3A), especially in wet sandy fields, secondary dunes, and marshlands, occasionally in disturbed areas. It is particularly frequent in recently burned areas.

##### Notes.

*Imperata brasiliensis* exhibits considerable morphological variation, ranging from very small individuals to much larger plants. The species is very similar to *I. tenuis* due to its contracted inflorescences with appressed basal branches. These two species have traditionally been distinguished based on plant height and inflorescence length, both of which are generally greater in *I. tenuis* (Gabel 1982; Flores 2001; Welker and Longhi-Wagner 2012; Peichoto and Welker 2023). In addition, *I. brasiliensis* typically has shorter and broader leaf blades than *I. tenuis*, with a less conspicuous pseudopetiole. However, individuals with intermediate morphological characteristics occur in populations of both species. Considering that polyploidy events are extremely common in Andropogoneae, and that at least one-third of the speciation events in the tribe have resulted from allopolyploidization (Estep et al. 2014; Welker et al. 2015, 2016), it is possible that the intermediate individuals represent natural hybrids between the two *Imperata* species. This hypothesis, however, requires testing through population-level molecular studies.

Field observations also suggest differences in growth habit between the species. *Imperata brasiliensis* tends to occur as sparsely distributed individuals connected by long rhizomes (Fig. 2A), whereas *I. tenuis* usually forms dense clumps (Fig. 2C). However, this feature is generally not evident in herbarium specimens because clumps are often disaggregated during preparation, as previously noted by Welker and Longhi-Wagner (2012).

The anatomical data presented here provide additional diagnostic characters for distinguishing the two species, as dumbbell-shaped guard cells on the adaxial leaf blade surface were observed only in *I. brasiliensis*. Moreover, cushion-hairs are present on both leaf surfaces in *I. brasiliensis*, but are absent in *I. tenuis* (see Comparative anatomy and micromorphology section below).

##### Selected specimens examined.

Brazil • **Bahia**: Itapebi, Rodovia Itapebi-Belmonte, 28 Sep. 1979, *L.A.M. Silva & J.L. Hage 623* (CEN, RB). **Distrito Federal**: Brasília, Reserva Ecológica do IBGE, 27 Jul. 1994, *F.C.A. Oliveira & M.L. Fonseca 36* (SP). **Espírito Santo**: Conceição da Barra, 04 Dec. 2014, *S. Rigo s.n*. (RB 657509). **Goiás**: Goiatuba, BR-153, 07 Sep. 1998, *R. Farias 112* (CEN). **Maranhão**: Monção, Basin of the Rio Turiaçu, Ka’apor Indian Reserve, 20 Jan. 1985, *W.L. Balée & B.G. Ribeiro 784* (K). **Mato Grosso**: Cáceres, Porto Limão, 19 Oct. 2005, *M.A. Carniello et al. 1002* (FLOR, HUFU). **Mato Grosso do Sul**: Miranda, Fazenda Bodoquena, 26 Oct. 1978, *A.C. Allem et al. 2167* (CEN, ICN). **Minas Gerais**: Marliéria, Parque Estadual do Rio Doce, Trilha para a lagoa Carioca, 18 Sep. 1998, *J.A. Lombardi 2407* (BHCB). **Paraná**: Guaraqueçaba, PR-405, 21 Dec. 2011, *C.A.D. Welker 381* (ICN). **Pernambuco**: Recife, Jan. 1975, *T. Sendulsky 1433* (SP). **Rio de Janeiro**: Mangaratiba, Ilha da Marambaia, Trilha para praia do Sino, 17 Aug. 2009, *C. Silva 101* (ICN). **Rio Grande do Sul**: Balneário Pinhal, Praia do Pinhal, 19 Jan. 2010, *C.A.D. Welker 287* (ICN). **Rondônia**: Porto Velho, Estação Experimental do Ministério da Agricultura, 27 Aug. 1970, *J.F.M. Valls 1213* (ICN). **Santa Catarina**: Garopaba, Porto da Cidade, 21 Oct. 1970, *R.M. Klein & A. Bresolin 8826* (FLOR, HUFU). **São Paulo**: Águas de Santa Bárbara, Estação Ecológica de Santa Bárbara, 13 Oct. 2016, *G.B. Assis et al. 276* (CEN, RB). **Tocantins**: Goiatins, BR-010, Alto Lindo-Goiatins, km 14, 21 Sep. 2009, *G. Pereira-Silva 14628* (CEN).

#### 2. Imperata contracta

Taxon classificationPlantaePoalesPoaceae

(Kunth) Hitchc., Rep. (Annual) Missouri Bot. Gard. 4: 146. 1893.

9F80ACFB-B9ED-5E76-BC7D-0A0226C0F5D5

Fig. 4

##### Basionym.

*Saccharum contractum* Kunth in Humb., Bonpl. & Kunth, Nov. Gen. Sp. 1: 182. 1816.

**Figure 4.**
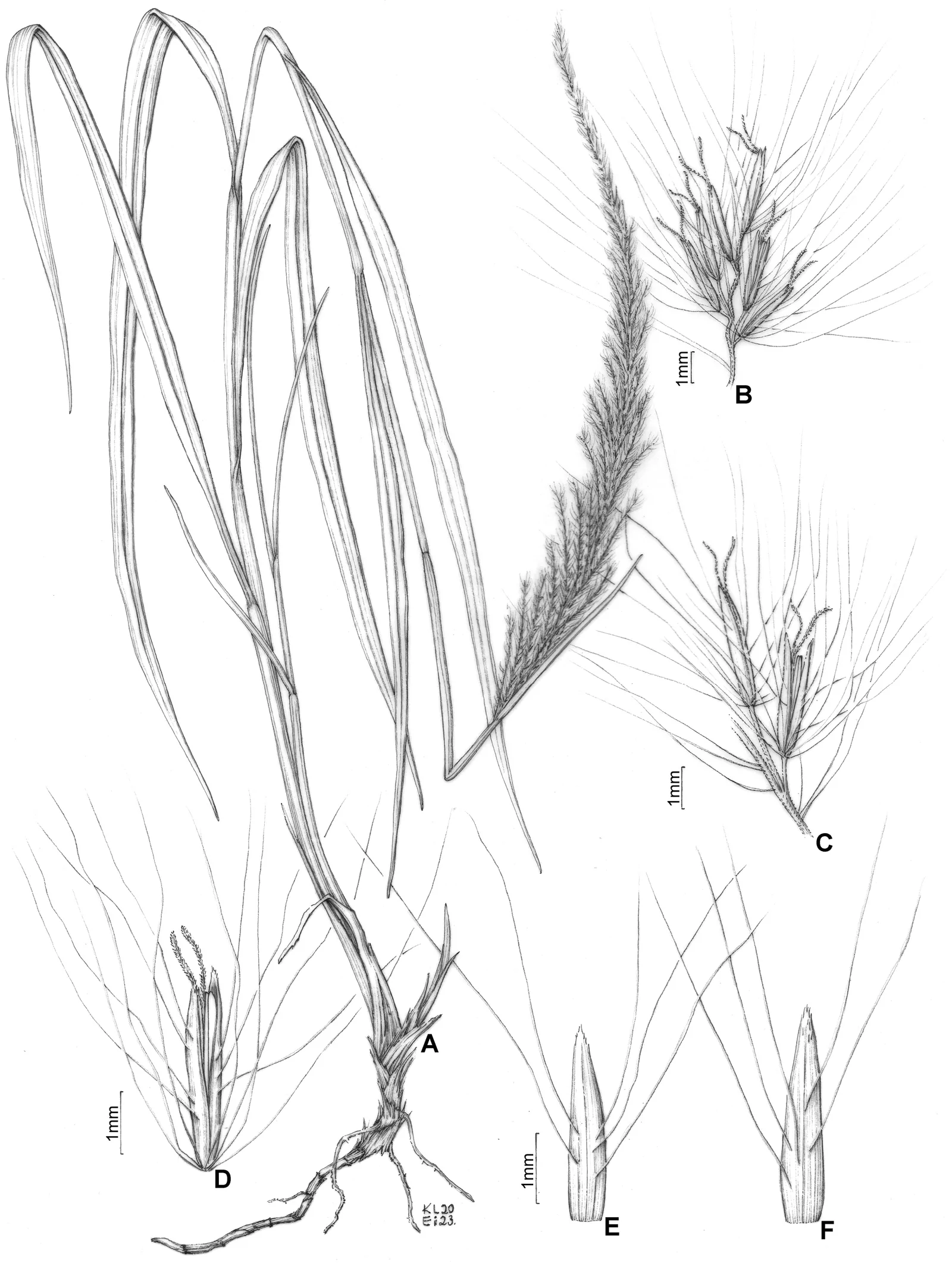
*Imperata contracta*. **A**. Habit; **B**. Portion of the inflorescence; **C**. Pair of spikelets; **D**. Spikelet showing stigma and anther; **E**. Lower glume; **F**. Upper glume. Based on *Valls 2667* (ICN).

##### Type.

Colombia • [*Crescit in humidis, calidissimis Regni Novogranatensis in ripa fluvii Magdalenae: item in temperatis montosis propter Cocollar, Turumiquiri et Caripe Cumanensium, alt. 450 hexap*.], May–Sep., *F.W.H.A. Humboldt & A.J.A. Bonpland 1600* (lectotype, designated here: P [P00669468 digital image!]; isolectotypes: BAA [BAA00002807 digital image!, fragm. ex P], P [P00128966 digital image!, P00128967 digital image!]).

##### Description.

Plants perennial, (85–)104–186 cm tall. Culms erect; nodes glabrous; internodes hollow. Leaf sheaths glabrous. Ligules membranous-ciliate, 0.5–2 mm long. Leaf blades (11–)16–85.5 × (0.4–)0.6–1.3 cm, linear-lanceolate, pseudopetiolate, with pseudopetiole reduced or not to the leaf midrib; abaxial surface glabrous; adaxial surface pilose, usually with tufts of trichomes near the ligule and occasionally with isolated trichomes along the blade; margins with prickle-hairs. Inflorescences (12–)24–51 cm long, subcontracted; basal branches (3.5–)4.7–10.1 cm long, divergent from the main axis. Rachis tough, bearing pairs of spikelets, both pedicellate, with pedicels of unequal length. Spikelets (2.1–)2.3–4(–4.3) mm long, muticous, with long silky white or pinkish-silvery trichomes; lower glume (1.8–)2–3.2(–3.6) mm long; upper glume (2–)2.1–3.3(–3.9) mm long. Lower floret sterile; lower lemma 1.3–2.4 mm long; lower palea absent. Upper floret bisexual; upper lemma 0.6–1.3(–1.5) mm long; upper palea 0.8–2.1 mm long. Stigmas 2, vinaceous, 1.3–3 mm long. Stamen 1; anther 1.2–2 mm long, yellow to orange-brown. Caryopsis 2–3 mm long.

##### Distribution and habitat.

Widely distributed from Mexico to Argentina and Brazil (Filgueiras 2003; Scrivanti et al. 2012). The species occurs mainly from central to southern Brazil (Fig. 3B), inhabiting wet grasslands, marshlands, and disturbed areas, and is occasionally invasive in plantations.

##### Notes.

*Saccharum contractum*, the basionym of *Imperata contracta*, was described by Kunth (1816), based on material collected by Friedrich W. H. A. von Humboldt and Aimé J. A. Bonpland in the Americas. However, the specimens analyzed were not explicitly cited in the protologue. According to *Taxonomic Literature II* (Stafleu and Cowan 1979: 693), “the Humboldt, Bonpland and Kunth types are mainly at P [Herbarium]”. Three specimens of the gathering *Humboldt & Bonpland 1600* were found at the Herbarium of Paris (P) and are treated here as original material of *Saccharum contractum*. Therefore, a lectotype designation is required under Art. 9.3 of the International Code of Nomenclature for algae, fungi, and plants (ICN; Turland et al. 2025). We hereby designate the specimen *Humboldt & Bonpland 1600* (P00669468) as the lectotype of *Saccharum contractum*, as it conforms to the original description and bears the label “Herbier Humboldt & Bonpland. Amérique Équatoriale”.

*Imperata contracta* is the most readily recognizable of the Brazilian species due to its large subcontracted inflorescences with divergent basal branches. These branches are usually longer and more conspicuous than in *I. brasiliensis* and *I. tenuis*, providing a consistent macromorphological character for identification. In anatomical terms, *I. contracta* differs from the other Brazilian species in the morphology of its leaf epidermal long and short cells (see details below), which provides additional support for its recognition.

##### Selected specimens examined.

Brazil • **Bahia**: Ilhéus, Área do CEPEC, 01 Jul. 1981, *J.L. Hage & H.S. Brito 1022* (CEPEC, K). **Minas Gerais**: Marliéria, Parque Florestal Estadual do Rio Doce, 18 May 1982, *E.F. Almeida 185* (K, MBM). **Paraná**: Morretes, Jacarehy, 10 May 1915, *P. Dusén 17024* (K). **Piauí**: Parnaguá, Rio São Joãozinho, 24 Apr. 1980, *G. Hatschbach et al. 42979* (MBM). **Rio de Janeiro**: Baixada de Jacarepaguá, à beira da Lagoa de Marapendi, 09 Apr. 1976, *D. Araújo 1076* (K, NY). **Rio Grande do Sul**: Taquara, 1 km em direção a Lageadinho, 08 Jul. 1973, *J.F.M. Valls 2667* (ICN). **Santa Catarina**: São Pedro de Alcântara, 14 May 1990, *M.H. Queiroz 217* (FLOR).

#### 3. Imperata tenuis

Taxon classificationPlantaePoalesPoaceae

Hack., Monogr. Phan. 6: 689. 1889.

D9283ADA-95FD-583A-AF51-C8C811933E67

Figs 2C, 2D, 5

##### Type.

Brazil • Minas Gerais: São João del-Rei, 1888, *A.F.M. Glaziou 17442* (lectotype, designated here: W [W1916-0034901 digital image!]; isolectotypes: K [K000632952!, K000632953!], P [P00740687 digital image!, P00740688 digital image!, P00740689 digital image!], US [US2942435!]).

**Figure 5.**
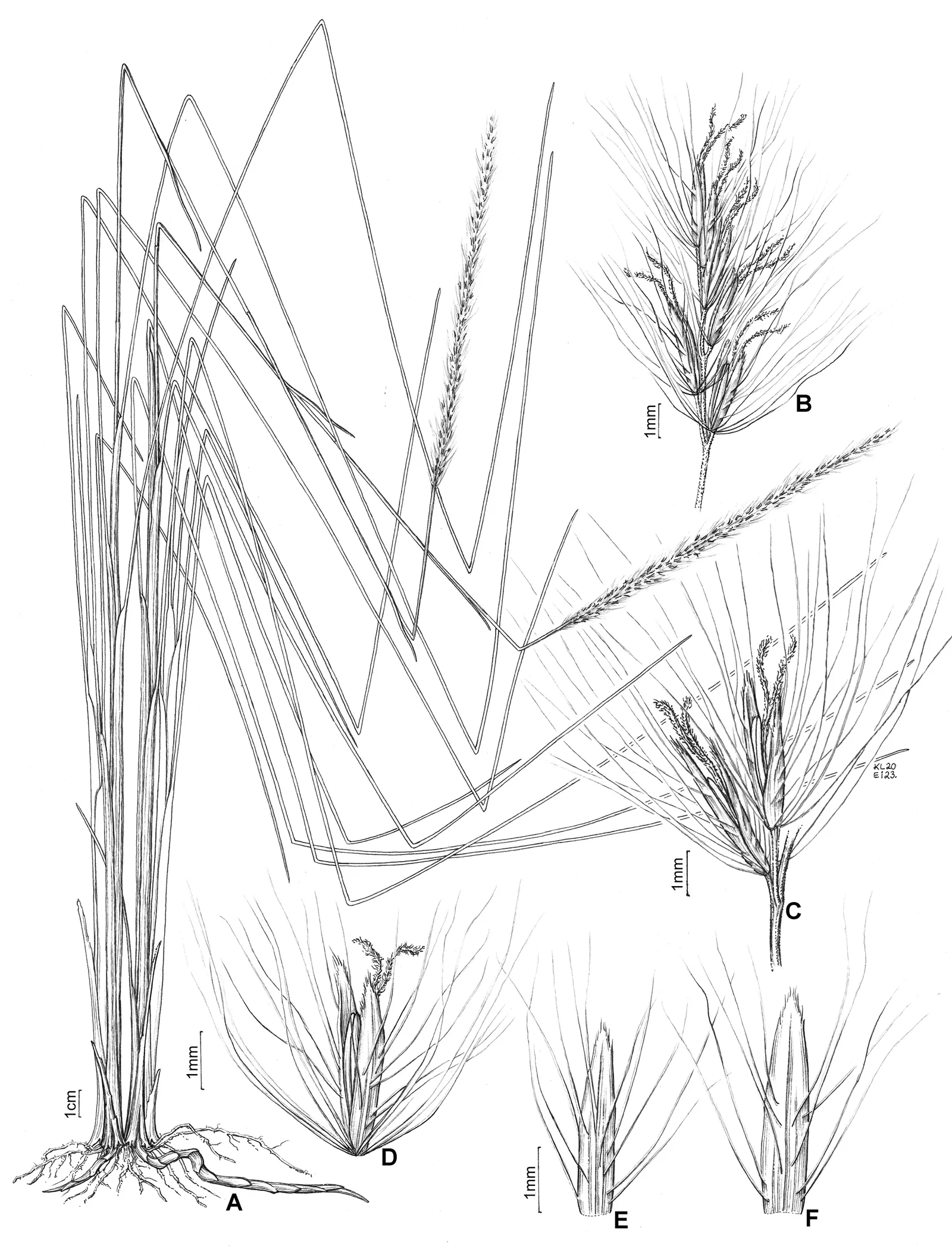
*Imperata tenuis*. **A**. Habit; **B**. Portion of the inflorescence; **C**. Pair of spikelets; **D**. Spikelet showing stigma and anther; **E**. Lower glume; **F**. Upper glume. Based on *Oliveira 402* (HUFU).

##### Description.

Plants perennial, (66–)81–177 cm tall. Culms erect; nodes glabrous; internodes hollow. Leaf sheaths glabrous. Ligules membranous-ciliate, 1–3 mm long. Leaf blades (11–)24–93(–97) × 0.1–0.6(–0.9) cm, linear, pseudopetiolate, with pseudopetiole generally reduced to the leaf midrib; abaxial surface glabrous; adaxial surface pilose, with a higher concentration of trichomes toward the apex and occasionally with tufts of trichomes near the ligule; margins with prickle-hairs. Inflorescences (10–)14–27 cm long, contracted; basal branches 1–5(–5.5) cm long, appressed to the main axis. Rachis tough, bearing pairs of spikelets, both pedicellate, with pedicels of unequal length. Spikelets 3–5 mm long, muticous, with long silky white trichomes; lower glume (2–)2.5–4.5 mm long; upper glume (2.7–)2.9–4.8 mm long. Lower floret sterile; lower lemma (1.5–)2–3(–3.6) mm long; lower palea absent. Upper floret bisexual; upper lemma 1–1.2 mm long; upper palea 0.9–1.5 mm long. Stigmas 2, vinaceous, (1–)2–3.5(–5) mm long. Stamen 1; anther (1.5–)2–3 mm long, yellow to orange-brown. Caryopsis 0.7–1.2 mm long.

##### Distribution and habitat.

Distributed in Argentina, Bolivia, Brazil, Ecuador, Paraguay, and Peru (Filgueiras 2003; Peichoto 2013). The species occurs mainly from central to southern Brazil (Fig. 3C), inhabiting wet grasslands, marshlands, and along watercourses and roadsides.

##### Notes.

Hackel (1889) described *Imperata tenuis* and cited the collection *Glaziou 17442* from Minas Gerais, Brazil, without indicating the herbarium where the material was deposited. As multiple duplicates of the gathering *Glaziou 17442* were found in the K, P, US, and W herbaria, all these specimens should be considered syntypes under Art. 9.6 of the ICN (Turland et al. 2025). We designate here the specimen deposited in the W herbarium (W1916-0034901) as the lectotype of *I. tenuis*, because it agrees with the original description of the species and its label bears the annotation “Ex herb. E. Hackel”.

*Imperata tenuis* is morphologically very similar to *I. brasiliensis*, and the overlap in plant height and inflorescence length has long complicated species delimitation (see comments under the latter species). Nevertheless, *I. tenuis* generally comprises taller plants with longer inflorescences and narrower leaves with a more conspicuous pseudopetiole, usually reduced to the midrib. The absence of cushion-hairs in the leaf epidermis provides an additional character that distinguishes *I. tenuis* from the other Brazilian species.

##### Selected specimens examined.

Brazil • **Goiás**: Santa Rita do Araguaia, on Rio Araguaia, 15 Apr. 1930, *A. Chase 12033* (RB, US). **Mato Grosso**: Poconé, Fazenda do Macaco, 07 Jun. 1992, *M. Schessl 107.4* (CEN). **Mato Grosso do Sul**: Corumbá, 6,6 km ao sul da Curva Leque e 14,6 km do rio Abobral, 27 Oct. 1986, *J.F.M. Valls 10353* (CEN). **Minas Gerais**: Uberlândia, Reserva Ecológica do Panga, 26 Jun. 2023, *B.L.S. Pinto & C.A.D. Welker 06* (HUFU). **Paraná**: rodovia 373, a 11 km de Guarapuava, 10 Dec. 1992, *Z. Rúgolo et al. 1655* (ICN). **Rio Grande do Sul**: São Gabriel, BR-290, próximo ao km 451, 15 Dec. 2009, *C.A.D. Welker 219* (HUCS, ICN, SMDB). **Santa Catarina**: Florianópolis, Ilha de Santa Catarina, Pântano do Sul, 23 Nov. 2009, *B. Toncic & A. Zanin 286* (FLOR). **São Paulo**: Votuporanga, Bairro Viradouro, 28 Jan. 1997, *A.D. Faria et al. 97/278* (ICN).

### Comparative anatomy and micromorphology

**Figure 6.**
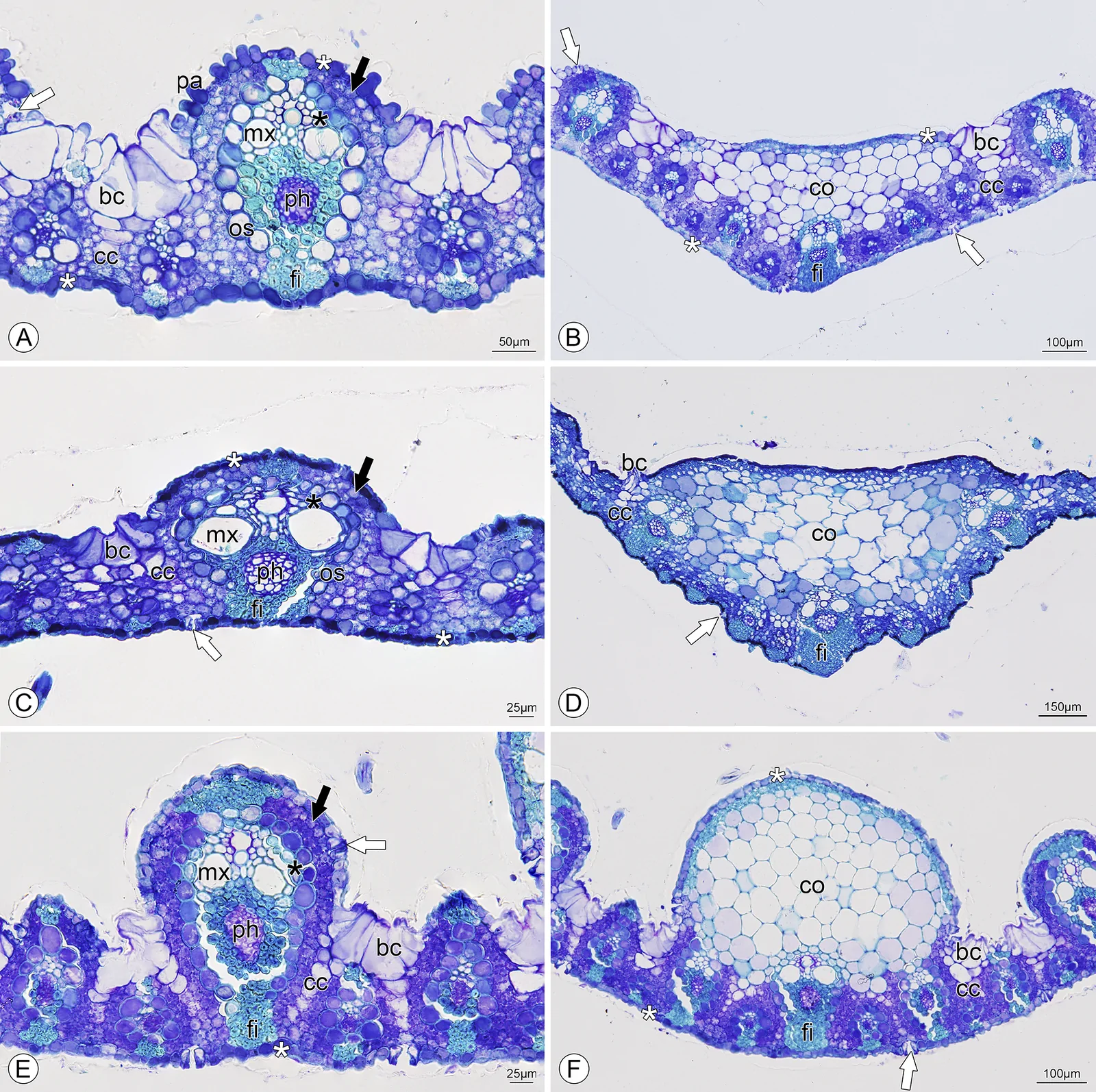
Leaf blades of *Imperata* in transverse sections. **A, B**. *I. brasiliensis*; **C, D**. *I. contracta*; **E, F**. *I. tenuis*. **A, C, E**. Detail of the mesophyll highlighting a first-order vascular bundle, followed by a fourth-order bundle on each side; **B, D, F**. Detail of the midrib. Note in B and D the keel formation in the midrib, and in F the projection of the cortex toward the adaxial surface. bc. bulliform cell; cc. colorless cell; co. cortex; fi. fiber; mx. metaxylem; os. outer bundle sheath; pa. papillae; ph. phloem. White asterisk: epidermis. Black asterisk: inner bundle sheath. White arrow: stomata. Black arrow: hypodermis.

**Figure 7.**
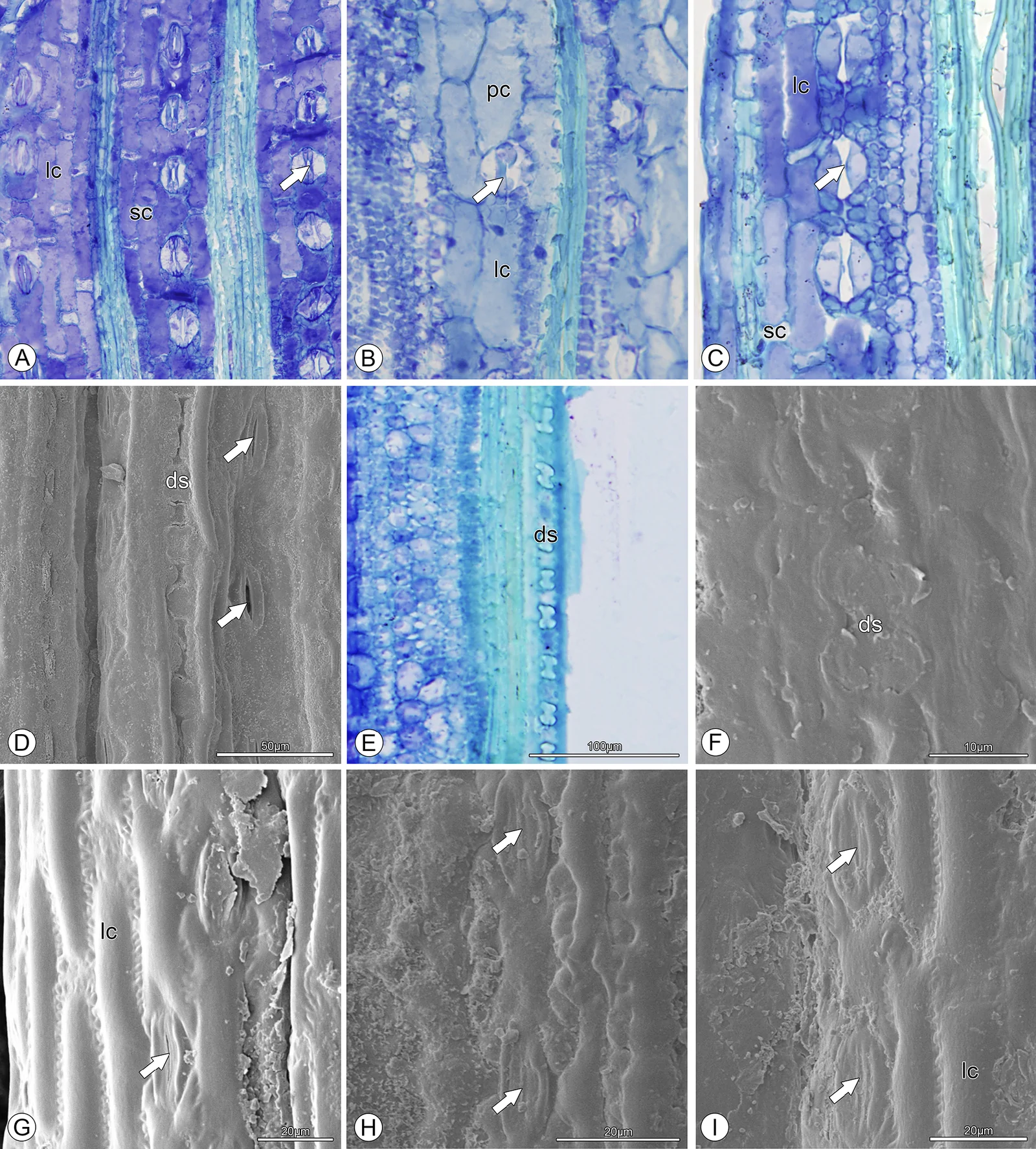
Leaf blades of *Imperata*, showing adaxial and abaxial epidermal surfaces in paradermal sections (**A–C, E**) and under scanning electron microscopy (**D, F–I**). **A, D, G**. *I. brasiliensis*; **B, E, H**. *I. contracta*; **C, F, I**. *I. tenuis*. Adaxial surface: **A, E, F, H**. Abaxial surface: **B–D, G, I**. **A–C**. Stomatal bands with long cells and short cells. Note in B the absence of rectangular short cells intercalating the long cells. **D–F**. Dumbbell-shaped short cells. **G–I**. Stomata under scanning electron microscopy. ds. dumbbell-shaped short cell; lc. rectangular long cell; pc. pentagonal long cell; sc. rectangular short cell. White arrow: stomata.

**Figure 8.**
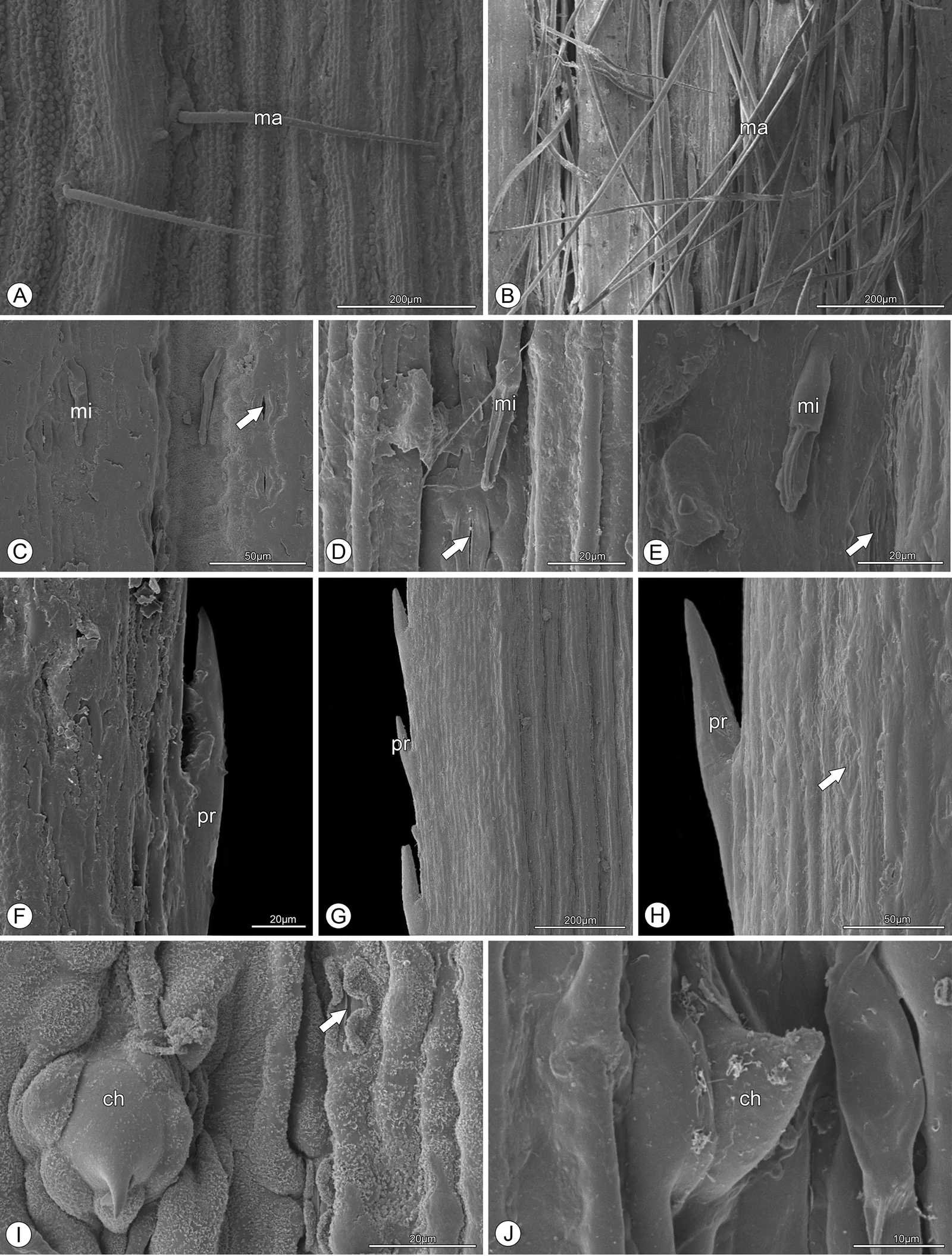
Leaf blades of *Imperata* under scanning electron microscopy, showing the different trichome types found in the genus. **C, F, I**. *I. brasiliensis*; **A, D, G, J**. *I. contracta*; **B, E, H**. *I. tenuis*. Adaxial surface: **A–C, E, I**. Abaxial surface: **D, F–H, J**. **A, B**. Macrotrichomes. **C–E**. Microtrichomes in intercostal regions. **F–H**. Prickle-hairs on the leaf margins. **I–J**. Cushion-hairs; **I**. Dumbbell-shaped guard cells. ch. cushion-hair; ma. macrotrichome; mi. microtrichome; pr. prickle-hair. White arrow: stomata.

**Figure 9.**
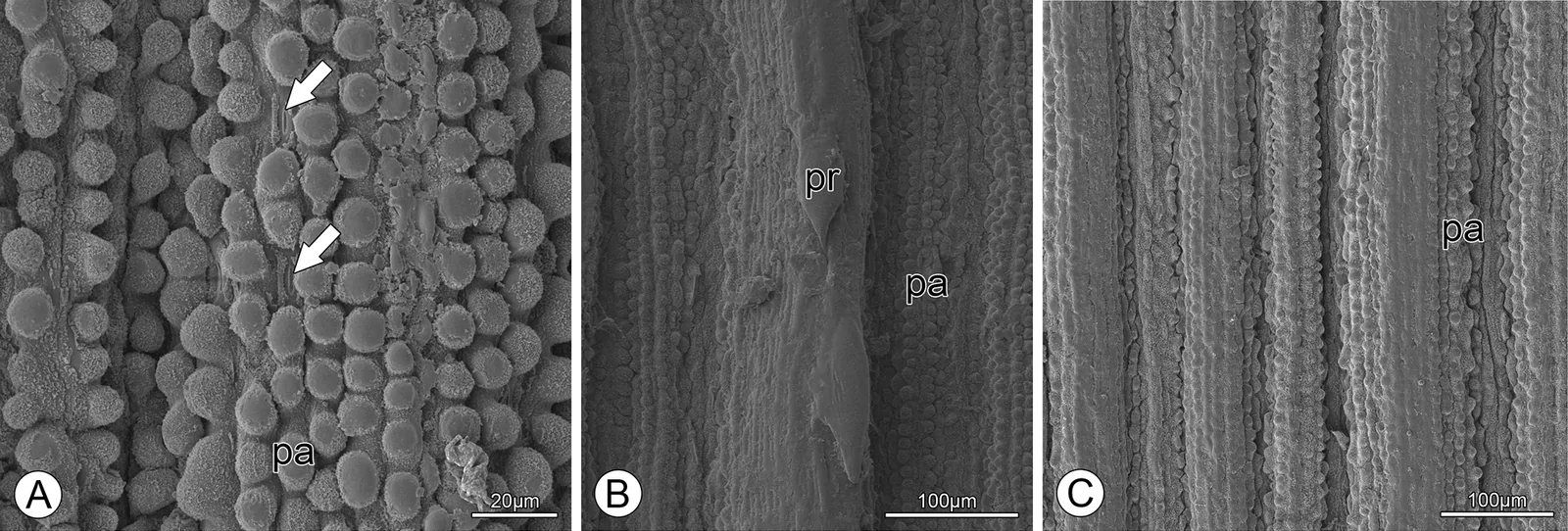
Leaf blades of *Imperata* under scanning electron microscopy, showing the adaxial epidermis with papillose cells. **A**. *I. brasiliensis*; **B**. *I. contracta*; **C**. *I. tenuis*. pa. papillae; pr. prickle-hair. White arrow: stomata.

The three Brazilian species of *Imperata* share the same general leaf blade organization, including projected costal regions (ribs) and intercostal furrows on the adaxial surface, amphistomatic leaves, paracytic stomata, papillate epidermal cells, radiate mesophyll, and collateral vascular bundles arranged in four orders, all with double lignified bundle sheaths (Figs 6, 7, 8, 9). Despite this overall uniformity, several anatomical and micromorphological characters differed among the species (Table 1). However, all observed characters were consistent across the specimens analyzed within each species.

**Table 1.** Comparison of the main anatomical and micromorphological characters of the leaf blades of the Brazilian species of *Imperata*.

**Character**	***Imperata brasiliensis***	***Imperata contracta***	***Imperata tenuis***
**Midrib**	U- or V-shaped keel projected toward the abaxial surface	U- or V-shaped keel projected toward the abaxial surface	No keel; cortex projected toward the adaxial surface
**Long cells**	Rectangular, with sinuous walls	Rectangular or pentagonal, with sinuous walls	Rectangular, with sinuous walls
**Short cells**	Rectangular or dumbbell-shaped	Dumbbell-shaped	Rectangular or dumbbell-shaped
**Stomata**	Elongated or dumbbell-shaped guard cells	Elongated guard cells	Elongated guard cells
**Prickle-hairs**	On leaf margins	On leaf margins and over major vascular bundles	On leaf margins
**Cushion-hairs**	Present on both surfaces, in the intercostal regions	Present on both surfaces, in the intercostal regions	Absent

In transverse section, the leaf blade showed adaxial ribs and furrows in all species (Fig. 6A, C, E). The midrib differed among species: in *Imperata brasiliensis* and *I. contracta*, it formed an abaxial keel (Fig. 6B, D), whereas in *I. tenuis* the cortex projected toward the adaxial surface and no abaxial keel was observed (Fig. 6F). Stomata occurred on both surfaces, although on the adaxial surface they were mainly associated with vascular bundles and bulliform cells (Figs 6A, 6C, 6E, 7G–I). All species had elongated guard cells, whereas dumbbell-shaped guard cells were observed only in *I. brasiliensis* (Fig. 8I).

The uniseriate epidermis had lignified walls, especially on the abaxial surface (Fig. 6A–F). Papillae occurred on the adaxial surface in all species, and bulliform cells were present in intercostal regions (Figs 6A, 6C, 6E, 9A–C). Rectangular long cells associated with stomatal bands were found in all species (Fig. 7A–C), whereas *I. contracta* also showed a distinct pentagonal long-cell type (Fig. 7B). Rectangular short cells occurred in *I. brasiliensis* and *I. tenuis* (Fig. 7A, C) but were absent from the stomatal bands of *I. contracta* (Fig. 7B). Dumbbell-shaped short cells were observed in all species (Fig. 7D–F).

Leaf appendages also varied among species. Macrotrichomes were present on the adaxial surface (Fig. 8A, B), microtrichomes occurred in intercostal regions (Fig. 8C–E), and prickle-hairs were found along the leaf margins of all species (Fig. 8F–H). In *I. contracta*, prickle-hairs were also observed in non-marginal regions (Fig. 9B). Cushion-hairs were present in *I. brasiliensis* and *I. contracta* (Fig. 8I, J) but were not observed in *I. tenuis*.

The mesophyll was homogeneous and radiate in all species, with one to two layers of chlorenchyma and an adaxial hypodermis (Fig. 6A, C, E). In the midrib, subepidermal fibers occurred on the adaxial side (Fig. 6B, D, F). Vascular bundles were arranged in four intercalated orders (Fig. 6A–F). In all species, the bundles had an outer and an inner lignified sheath, and lignified bundle sheath extensions usually connected the vascular bundles to the epidermis (Fig. 6A–F). In the midrib, first-order bundles lacked the outer sheath (Fig. 6B, D, F). *Imperata contracta* had three first-order bundles in the midrib region (Fig. 6D), whereas *I. brasiliensis* and *I. tenuis* had only one (Fig. 6B, F).

**Figure 10.**
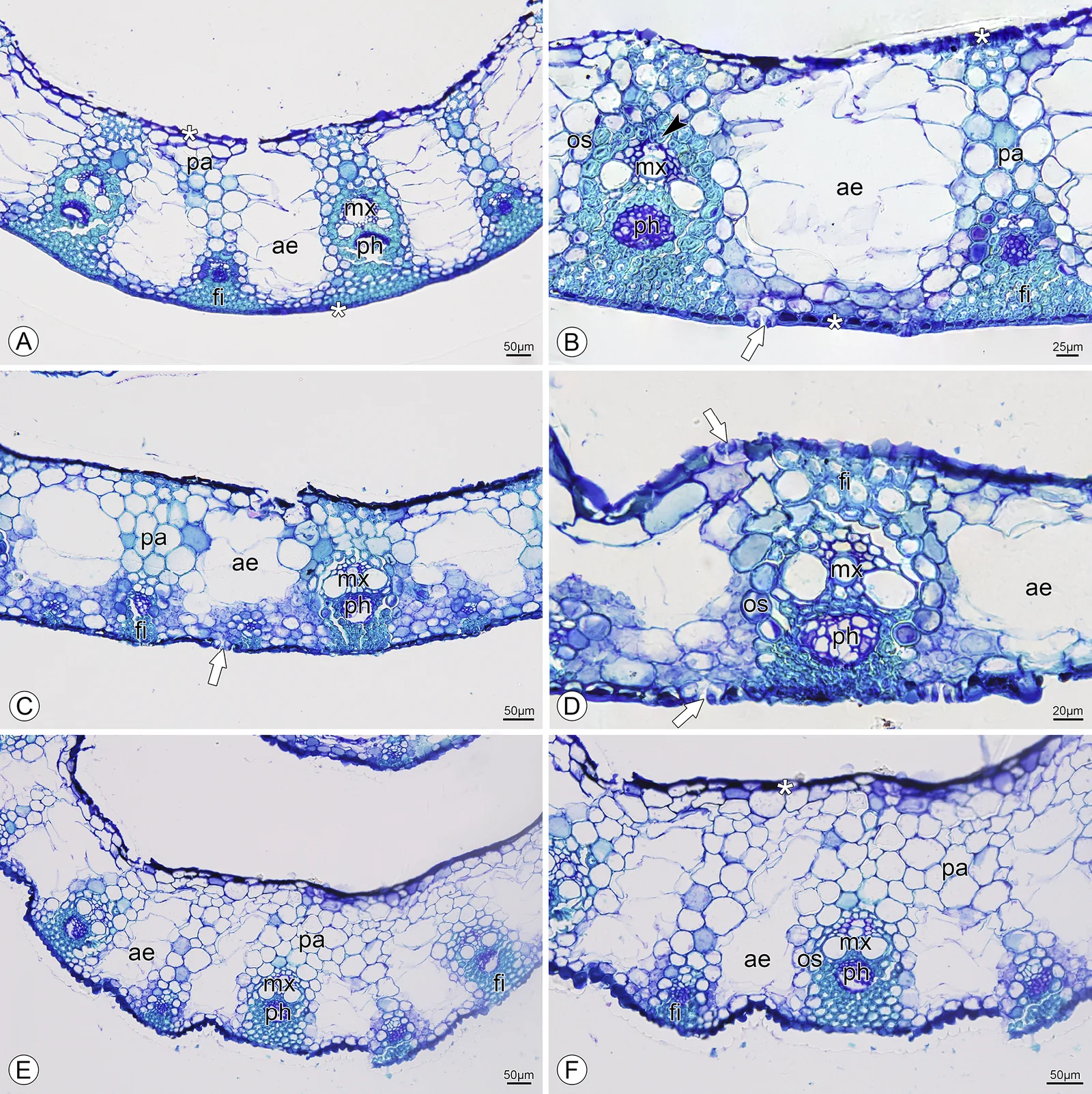
Leaf sheaths of *Imperata* in transverse sections. **A, B**. *I. brasiliensis*; **C, D**. *I. contracta*; **E, F**. *I. tenuis*. ae. aerenchyma; fi. fibers; mx. metaxylem; os. outer bundle sheath; pa. parenchyma; ph. phloem. Arrowhead: inner bundle sheath. White asterisk: epidermal cells with thickened walls. White arrow: stomata.

Leaf sheath anatomy was more uniform among species (Fig. 10A–F). The sheath lacked ribs and a differentiated midrib and showed a uniseriate epidermis with thickened walls. Stomata occurred on both surfaces but were more frequent on the abaxial surface. Large aerenchyma spaces were present among the vascular bundles, and only two bundle types were recognized (Fig. 10A–F).

**Figure 11.**
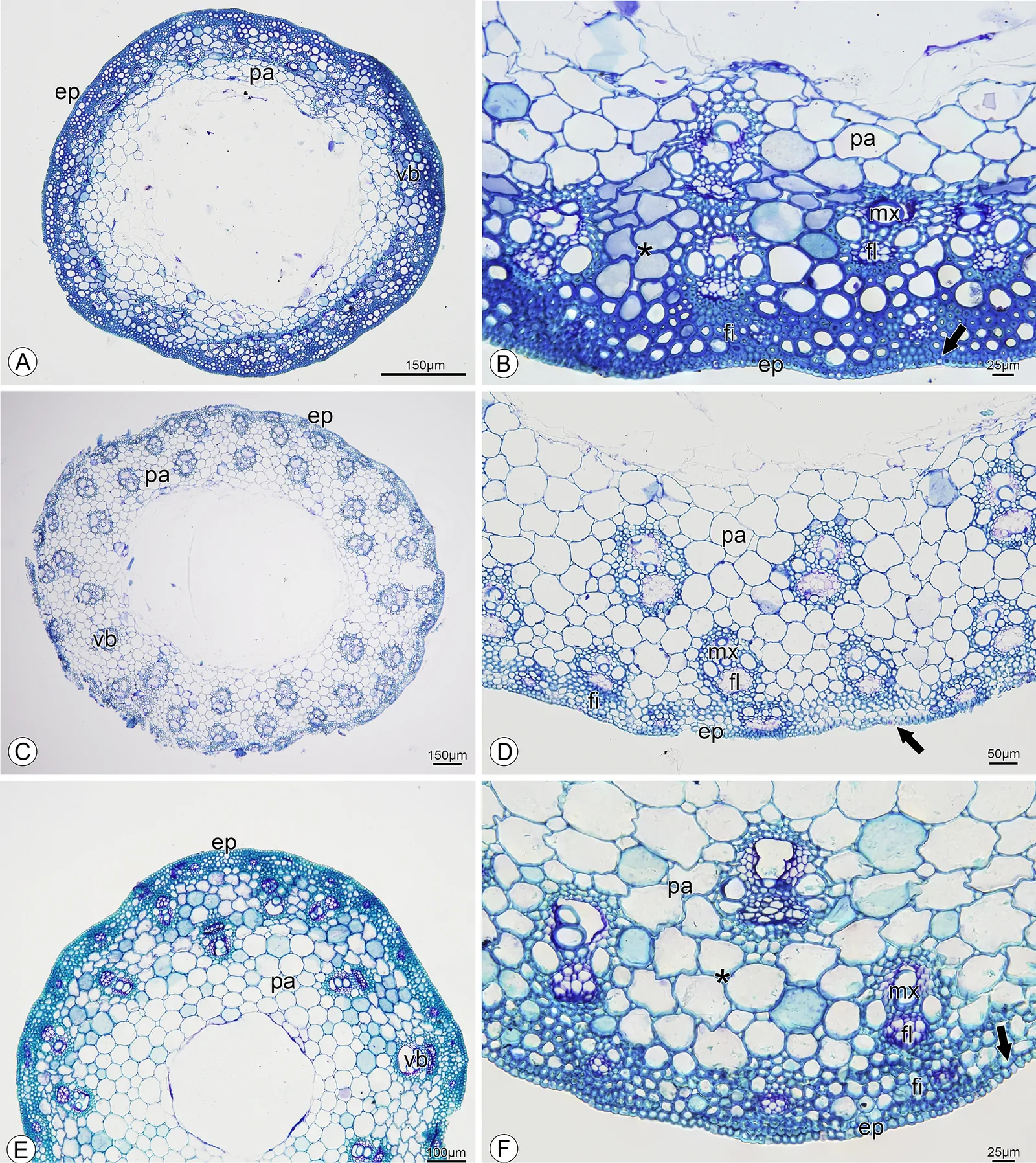
Culms of *Imperata* in transverse sections. **A, B**. *I. brasiliensis*; **C, D**. *I. contracta*; **E, F**. *I. tenuis*. ep. epidermis; fi. fibers; mx. metaxylem; pa. parenchyma with intercellular spaces; pc. pith cavity; ph. phloem; vb. vascular bundle. Black asterisk: parenchyma cells with strongly thickened walls. Black arrow: short cell.

Culm anatomy was also similar across species (Fig. 11A–F). Culms were cylindrical and hollow, with a uniseriate epidermis of small, thick-walled cells. Short epidermal cells were present, but stomata, papillae, and trichomes were not observed. Internally, the culm was differentiated into cortex, vascular system, and pith, with parenchymatic cells showing markedly thickened walls (Fig. 11A–F).

## Discussion

The confirmation of *Imperata brasiliensis*, *I. contracta*, and *I. tenuis* for Brazil is consistent with previous taxonomic treatments of the genus (e.g., Flores 2001; Welker and Longhi-Wagner 2012; Flora e Funga do Brasil 2026). By contrast, *I. minutiflora*, previously reported for Brazil by Filgueiras (2003) and Flora e Funga do Brasil (2026) without citation of examined specimens, was not confirmed and is therefore excluded from the Brazilian flora. This result concurs with published accounts of the distribution of *I. minutiflora*, which restrict the taxon to Argentina, Bolivia, Ecuador, and Peru (Gabel 1982). The species is distinguished primarily by its very small spikelets, measuring 1.5–2 mm long (Scrivanti et al. 2012), which are smaller than those of the three species confirmed here for Brazil.

From an anatomical perspective, the Brazilian species of *Imperata* share a leaf structural pattern consistent with previous anatomical studies of the genus and other members of the tribe Andropogoneae. The occurrence of papillate epidermal cells, radiate mesophyll, collateral vascular bundles arranged in four orders, and double lignified bundle sheaths agrees with the pattern described for *I. cylindrica* by Renvoize (1982) and by subsequent studies on this species (Ahmad et al. 2010; Ullah et al. 2011; Nazir et al. 2013). More broadly, foliar characters such as epidermal cell types, trichomes, and bundle sheath organization have proven taxonomically informative within Andropogoneae, both in anatomical studies (Peichoto 2003; Ahmad et al. 2010; Ullah et al. 2011; Nazir et al. 2013; Farr et al. 2024) and in micromorphological investigations using scanning electron microscopy (Hilu 1984; Dávila and Clark 1990; Khan et al. 2017). Our results therefore reinforce previous findings that leaf anatomy and micromorphology provide valuable evidence for comparative studies among taxa within the tribe.

Within this shared structural pattern, the main interspecific contrasts were concentrated in the leaf blade, especially in epidermal and micromorphological characters. Among these, the occurrence of stomata with dumbbell-shaped guard cells in *I. brasiliensis*, the distinctive morphology of the long and short cells in *I. contracta*, and the absence of cushion-hairs in *I. tenuis* are particularly noteworthy. These characters complement macromorphology, which in *Imperata* often shows overlap in quantitative attributes such as plant height, leaf width, and inflorescence length (Gabel 1982; Flores 2001; Welker and Longhi-Wagner 2012; Peichoto 2013; Peichoto and Welker 2023).

The anatomical and micromorphological differences observed here between *I. brasiliensis* and *I. tenuis* are particularly important, as these species have long been considered difficult to distinguish based on macromorphology alone (Welker and Longhi-Wagner 2012; Peichoto 2013). This challenge is further reflected in recent phylogenetic analyses of Neotropical *Imperata*, which recovered the two species as closely related but without clear resolution between them, highlighting the need for complementary evidence to improve their delimitation (Cordobés et al. 2021). In the material examined here, specimens of *I. brasiliensis* were generally smaller, with shorter inflorescences and broader leaves, whereas those of *I. tenuis* were generally taller, with longer inflorescences, narrower leaves, and a more conspicuous pseudopetiole, often reduced to the midrib. These macromorphological differences are now further supported by anatomical and micromorphological traits, such as the presence of cushion-hairs and dumbbell-shaped guard cells in *I. brasiliensis*, both of which are absent in *I. tenuis*. Therefore, this study provides additional diagnostic characters that contribute to the distinction between these two species and help clarify this taxonomic issue. Nevertheless, further studies, particularly those involving cytogenetics and population-level molecular analyses, are needed to evaluate the possibility of natural hybridization between these species.

In contrast to the leaf blade, the anatomy of the leaf sheath and culm showed comparatively little variation among species and therefore contributed less to species delimitation. The leaf sheath consistently exhibited large aerenchyma spaces, two types of vascular bundles, and a thickened epidermis, whereas the culm was characterized by hollow internodes and thick-walled parenchymatic cells in all analyzed species. Although these features are relevant for the structural characterization of *Imperata* in Brazil, they have lower discriminatory value than the characters observed in the leaf blade.

## Supplementary Material

XML Treatment for Imperata brasiliensis

XML Treatment for Imperata contracta

XML Treatment for Imperata tenuis

## Additional information

### Artificial Intelligence (AI) use

The authors accept full responsibility for the content of the manuscript, including the disclosure of any use of AI.

AI was used only for language editing and stylistic refinement.

### Author ORCIDs

B.L.S. Pinto https://orcid.org/0000-0001-7472-523X

C.A.D. Welker https://orcid.org/0000-0001-6347-341X

J. Marzinek https://orcid.org/0000-0002-4482-0655

### Data availability

All of the data that support the findings of this study are available in the main text or Supplementary Information.

## Supplementary materials

Supplementary material 1 List of specimens examined for the taxonomic reassessment of Brazilian *Imperata* Data type docx https://binary.pensoft.net/file/1779705This dataset is made available under the Open Database License (http://opendatacommons.org/licenses/odbl/1.0/). The Open Database License (ODbL) is a license agreement intended to allow users to freely share, modify, and use this Dataset while maintaining this same freedom for others, provided that the original source and author(s) are credited. Bianca L. S. Pinto, Cassiano A. D. Welker, Juliana Marzinek
