# Supplementary material for: Anatomy and micromorphology of Imperata (Poaceae, Andropogoneae) from Brazil and their taxonomic significance
Source: PhytoKeys. 2026 Sep 18;279:155–78. doi: 10.3897/phytokeys.279.207098 (PMC13615398; doi:10.3897/phytokeys.279.207098)
Supplement: Supplementary file 1 [file phytokeys-279-155_article-207098__-s001.docx]

**Supplementary Material 1**

**Examined specimens of *Imperata* (Poaceae, Andropogoneae)**

This file contains the complete list of specimens examined for the taxonomic reassessment of Brazilian *Imperata.* Selected specimens cited in the main text are complemented here by the full voucher list, arranged by species in alphabetical order.

***Imperata brasiliensis* Trin.**

**Specimens examined.** BRAZIL. **Bahia:** Camumu, BR-101, 9 km a leste do entroncamento, 19 Sep 1988, *LA Mattos Silva et al.* *2530* (MBM 126483). Itapebi, Rodovia Itapebi-Belmonte, próximo ao Posto, entre Nova Itapebi e a BR-101, 28 Sep 1979, *LAM Silva & JL Hage 623* (CEN 4917, RB 198166). **Distrito Federal:** Brasília, Reserva Ecológica do IBGE, 27 Jul 1994, *FCA Oliveira & ML Fonseca 36* (SP 367066). Brasília, Reserva Ecológica do IBGE, 28 Oct 2005, *MA Silva 5765* (CEN 69920). **Espírito Santo:** Conceição da Barra, 04 Dec 2014, *S Rigo s.n.* (RB 657509). **Goiás:** Estrela do Norte, Rodovia Anápolis-Belém, km 386, 04 Oct 1980, *L Coradin et al. 3517* (CEN 6659). Goiatuba, BR-153, 07 Sep 1998, *R Farias 112* (CEN 35799). **Maranhão:** Monção, Basin of the Rio Turiaçu, Ka'apor Indian Reserve, 20 Jan 1985, *WL Balée & BG Ribeiro 784* (K 1068645). **Mato Grosso:** Cáceres, Porto Limão, 19 Oct 2005, *MA Carniello et al. 1002* (FLOR 36390, HUFU 84832). Fazenda Santa Lúcia, 04 Aug 1973, *L Arzivenco 621* (ICN 034439). Poconé, 19,1 km ao sul do portão de entrada, estrada Transpantaneira, 23 Oct 1985, *JFM Valls et al. 9325* (CEN 70352). 12 km ao sul de Poconé, 13 Oct 1992, *M Schessl 2* (CEN 51515). **Mato Grosso do Sul:** Corumbá, 10 km desde Morrinho, em direção a Corumbá, ao longo da BR-262, 11 Oct 1985, *JFM Valls et al. 9120* (CEN 70250). Miranda, Fazenda Bodoquena, 26 Oct 1978, *AC Allem et al. 2167* (CEN 2371, ICN 047841). **Minas Gerais:** Baependi, estrada para São Tomé das Letras, 20 Jan 1995, *HM Longhi-Wagner 2753* (ICN 110515). Belo Horizonte, Campus da UFMG, Escola de Medicina Veterinária, Oct 1979, *JA Oliveira* 162 (BHCB 701). Belo Horizonte, Aug 1986, *Andréia s.n.* (BHCB 16248). Marliéria, Parque Estadual do Rio Doce, Trilha para a lagoa Carioca, 18 Sep 1998, *JA Lombardi 2407* (BHCB 43104). Olaria, 07 Sep 1988, *L Krieger & M Bragger 22547* (MBM 254388). Parque Nacional Grande Sertão Veredas, 21 Jul 1994, *FCA Oliveira & ML Fonseca 14* (SP 358744). São Tomé das Letras, 06 Dec 1997, *HM Longhi-Wagner et al. 4052* (ICN 126703). Serra da Lapa, Nov 1823, *L Riedel 1016* (K 632954, LE 639). Tiradentes, Serra de São José, 11 Oct 1999, *HM Longhi-Wagner & RJV Alves 7056* (ICN 186669). **Paraná:** Arapoti, 18 Oct 1966, *J Lindeman & H Haas 3122* (MBM 4304). Balsa Nova, Ponte dos Arcos, 01 Nov 2005, *C Kozera & A Sanches 2527* (MBM 319253). Cerro Azul, Serra do Canha, *G Hatschbach 8418* (MBM 26004). Guaraqueçaba, PR-405, 21 Dec 2011, *CAD Welker 381* (ICN 182077). Guaratuba, Morro Brejatuba, *G Hatschbach 4451* (MBM 26003). Palmas, Santo Agostinho, 06 Dec 1971, *G Hatschbach et al. 28290* (MBM 19505). Paranaguá, Pontal do Sul, 17 Jan 1982, *LT Dombrowski 12666* (MBM 224708). Paranaguá, Pontal do Sul, 08 Sep 1982, *LT Dombrowski 13345* (MBM 224676). Paranaguá, Ilha do Mel, Praia das Conchas, 09 Mar 1985, *SM Silva & RM Britez s.n.* (MBM 224762). Paranaguá, Pontal do Sul, 21 Apr 1989, *LT Dombrowski 13862* (MBM 224677). Ponta Grossa, caminho a Tibagí, a 13 km na BR-376, 12 Dec 1992, *Z Rúgolo et al. 1681* (ICN 101159). Pontal do Paraná, Balneário Shangri-lá, 10 Sep 2006, *F Marinero 53* (MBM 320576). São Jerônimo da Serra, Reserva Indígena São Jerônimo, 05 Sep 2002, *KLVR de Sá et al. 307* (MBM 304163). **Pernambuco:** Recife, Jan 1975, *T Sendulsky 1433* (SP 175456). **Rio de Janeiro:** Mangaratiba, Ilha da Marambaia, Trilha para praia do Sino, 17 Aug 2009, *C* *Silva 101* (ICN 162834). Piraí, Estrada para Barra do Piraí, 10 Aug 1990, *M Marchi 111* (ICN 089266). Rio de Janeiro, Pedra da Gávea, deserto campado em frente ao caminho dos Olhos, próximo ao Largo do Caneca, cerca de 600 m.s.m., 16 Sep 1973, *P Caracuta 1661* (RB 162373). Rio de Janeiro, Restinga de Jacarepaguá próximo a Pedra de Itaúna, 10 Oct 1987, *LC Giordano & LS Sarahyba 380* (CEN 82189). **Rio Grande do Sul:** Balneário Pinhal, Praia do Pinhal, 19 Jan 2010, *CAD Welker 287* (ICN 168826). Osório, 13 Sep 2015, *J Gaio et al. 457* (FURB 60244). Palmares do Sul, Quintão, 16 Nov 1995, *V Gonçalves 91* (FLOR 37008). Palmares do Sul, Quintão, Dunas Altas, 26 Nov 2011, *E Valduga 157* (FURB 37734, MBM 388613). Palmares do Sul, Praia do Quintão, margem da lagoa do Cipó, 24 Sep 2012, *F Gonzatti 593* (FURB 44266, MBM 391057). Rio Grande, Estação Ecológica do Taim, em frente à sede da Estação, 10 Jul 1986, *JA Jarenkow 381* (FLOR 19232). Santa Vitória do Palmar, Estação Ecológica do Taim, litoral atlântico em frente à Fazenda Caçapava, 04 Nov 1985, *JA Jarenkow et al. 273* (FLOR 19638). São Leopoldo, 27 Nov 1935, *C Orth s.n.* (SP 51182). Tupanciretã, 4 km ao norte do rio Ivaí, ao longo da BR-158, 06 Dec 1986, *JFM Valls 10715* (CEN 115860). **Rondônia:** Porto Velho, Estação Experimental do Ministério da Agricultura, 27 Aug 1970, *JFM Valls 1213* (ICN 024263). Porto Velho, margem direita do Rio Madeira, 6 km a oeste do porto da balsa para Vila São Lourenço, 24 Oct 2020, *MF Simon et al. 3935* (CEN 116730). **Santa Catarina:** Campos Novos, 15 Mar 1984, *G Hashimoto 10818* (SP 235392). Florianópolis, Ilha de Santa Catarina, 05 Oct 1964, *RM Klein et al. 5847* (FLOR 5734). Florianópolis, Ilha de Santa Catarina, Jurerê, 07 Oct 1964, *RM Klein et al.* *5923* (FLOR 5739). Florianópolis, Ilha de Santa Catarina, Rio Vermelho, 05 Oct 1984, *ML Souza et al. 273* (FLOR 21884, MBM 164758). Florianópolis, Ilha de Santa Catarina, Rio Vermelho, 16 Nov 1984, *FA Silva et al. 188* (FLOR 21887, MBM 164759). Florianópolis, Ilha de Santa Catarina, Pontal do Daniela, 02 Oct 1987, *ML Souza & AC Araújo 972* (FLOR 17932, MBM 131025). Florianópolis, Ilha de Santa Catarina, Pontal do Daniela, 23 Feb 1988, *ML Souza et al. 992* (FLOR 17904, MBM 131024). Florianópolis, Ilha de Santa Catarina, Dunas da Joaquina, 10 May 1991, *MH Queiroz 416* (FLOR 29614). Florianópolis, Ilha de Santa Catarina, Jurerê Internacional, 01 Sep 1993, *SC Boechat s.n.* (FLOR 26132, ICN 101767). Florianópolis, Ilha de Santa Catarina, Parque Municipal das Dunas da Lagoa da Conceição, 05 Mar 2004, *TB Guimarães & DB Falkenberg 612* (FLOR 35454). Florianópolis, Ilha de Santa Catarina, Parque Municipal das Dunas da Lagoa da Conceição, 25 May 2004, *TB Guimarães & DB Falkenberg* *1115* (FLOR 35452). Florianópolis, Ilha de Santa Catarina, Parque Municipal das Dunas da Lagoa da Conceição, 22 Aug 2004, *TB Guimarães & DB Falkenberg 612* (FLOR 35453). Florianópolis, Ilha de Santa Catarina, Parque Municipal das Dunas da Lagoa da Conceição, 28 Jul 2005, *TB Guimarães & DB Falkenberg 470* (FLOR 35451, HUFU 84836). Florianópolis, Ilha de Santa Catarina, Ingleses, 23 Jan 2009, *A Zanin & BH Santos 1577* (FLOR 37139). Florianópolis, Ilha de Santa Catarina, Ingleses, dunas entre Ingleses e Santinho, 11 May 2009*, B Toncic & A Zanin 224* (FLOR 37309). Florianópolis, Ilha de Santa Catarina, Carianos, Fazenda da Ressacada, 16 Nov 2009, *B Toncic & A Zanin 260* (FLOR 37444). Florianópolis, Ilha de Santa Catarina, Dunas da Praia dos Ingleses, 08 Jan 2010, *FB Guimarães 1144* (FLOR 37670). Florianópolis, Ilha de Santa Catarina, Parque Estadual do Rio Vermelho - PAERVE, 07 Nov 2012, *A Nuernberg 816* (FLOR 51484). Florianópolis, Ilha de Santa Catarina, Parque Estadual do Rio Vermelho - PAERVE, 21 Nov 2012, *A Nuernberg 914* (FLOR 51699). Florianópolis, Aterro da Costeira, 12 Sep 2013, *GN Hassemer & R Trevisan* *718* (FLOR 51211). Florianópolis, Aterro da Baía Sul, 08 Oct 2014, *LA Funez 3519* (FURB 45027). Florianópolis, 28 Jun 2015, *LA Funez 4427* (FURB 48166). Florianópolis, 28 Jul 2015, *LA Funez 4443* (FURB 48182). Garopaba, Porto da Cidade, 21 Oct 1970, *RM Klein & A Bresolin* *8826* (FLOR 5498, HUFU 84829). Garopaba, Siriú, 16 Mar 1971, *A Bresolin 177* (FLOR 4726). Garopaba, Siriú, 10 Aug 1971, *A Bresolin 275* (FLOR 5245). Grão-Pará, SC-439, encosta superior da face leste da Serra do Corvo Branco, 24 Apr 1997, *DB Falkenberg 9934* (FLOR 56542). Içara, Balneário Rincão, 09 Dec 2010, *A Korte & MJ Rigor Jr 5572* (FLOR 40949). Içara, Balneário Rincão, 06 Nov 2011, *EB Dalmolim* (FLOR 42616). Itajaí, Morro da Cruz, 17 Sep 1961, *RM Klein 2514* (FLOR 578). Navegantes, Meia Praia, 06 Nov 2012, *LA Funez 1157* (FURB 39501). São Francisco do Sul, Vila da Glória, 20 Jan 2007, *JZ Beger 590* (MBM 325222). Timbé do Sul, Serra da Rocinha, 13 Jun 2009, *M Verdi et al.* 2326 (FURB 12765). **São Paulo:** Águas de Santa Bárbara, Estação Ecológica de Santa Bárbara, 13 Oct 2016, *GB Assis et al. 276* (CEN 100914, RB 675183). Mogi-Guaçu, Estação Biológica de Mogi-Guaçu, IBT, 29 Oct 2010*, RT Shirasuna & SAC Chiea 2900* (SP 425874). Santo Antônio do Pinhal, 09 Dec 1997, *HM Longhi-Wagner* & *Witten 2786* (ICN 110550). São Paulo, Reserva Biológica, Parque Estadual das Fontes do Ipiranga, 01 Nov 1974, *T Sendulsky 1430* (CEN 52523). **Tocantins:** Goiatins, BR-010, Alto Lindo-Goiatins, km 14, 21 Sep 2009, *G Pereira-Silva 14628* (CEN 99852).

***Imperata contracta* (Kunth) Hitchc.**

**Specimens examined.** BRAZIL. **Bahia:** Ilhéus, Área do CEPEC, km 22 da Rodovia Ilhéus/Itabuna, 01 Jul 1981, *JL Hage & HS Brito 1022* (CEPEC 24064, K 4777). **Minas Gerais:** Formoso, Parque Nacional Grande Sertão Veredas, entre Vereda do Sumidouro e Vereda da Mutuca, 09 Oct 1988, *TS Filgueiras 2385* (SP 258149). Marliéria, Parque Florestal Estadual do Rio Doce, 18 May 1982, *EF Almeida 185* (K 1068652, MBM 162988). **Paraná:** Morretes, Jacarehy, 10 May 1915, *P Dusén 17024* (K 1068650). **Piauí:** Paranaguá, Rio São Joãozinho, 24 Apr 1980, *G Hatschbach et al. 42979* (MBM 67591). **Rio de Janeiro:** Baixada de Jacarepaguá, à beira da Lagoa de Marapendi, 09 Apr 1976, *D Araújo 1076* (K 1068656, NY 649916). **Rio Grande do Sul:** Feliz, 19 Jun 1971, *ML Lorscheitter & LRM Baptista s.n.* (ICN 033395). Osório, Estrada Expressa de Maquiné, 22 Apr 1974, *O Ruckiem s.n.* (ICN 025599). Osório, Rodovia de Morro Alto a Capão da Canoa, 24 Apr 1974, *JFM Valls et al. 3291* (CEN 4048, ICN 025616). Taquara, 1 km em direção a Lageadinho, 08 Jul 1973, *JFM Valls 2667* (ICN 024081). Torres, entre Vila São João e Praia Grande, 29 Dec 1970, *JFM Valls & L Arzivenco 1384* (ICN 010149). **Santa Catarina:** Ascurra, Morro do Oitenta, 19 Feb 2015, *LA Funez 4249* (FURB 47179). Florianópolis, Bairro Trindade, 15 Mar 2014, *LA Funez 2769* (FURB 43897). Rodeio, 25 Aug 2012, *LA Funez 835* (FURB 39002). São Pedro de Alcântara, 14 May 1990, *MH Queiroz 217* (FLOR 29624, FLOR 38078). COLOMBIA. May–Sep, *FWHA Humboldt & AJA Bonpland 1600* (BAA 2807, P 128966, P 128967, P 669468).

***Imperata minutiflora* Hack.**

**Specimens examined.** ARGENTINA. **Jujuy:** Ledesma, Puesto Caimancito de YPF, 07 Mar 1987, *FO Zuloaga & O Morrone 3033* (K 3913591). BOLIVIA. **La Paz:** Sud Yungas, 10 km East of new Alto Beni bridge, 09 Mar 1987, *S Renvoize 4723* (K 1289191). ECUADOR. **Loja:** Catamayo, 17 Feb 1987, *I Grignon 84254* (K 3913554). PERU. **Cajamarca:** 83 km from Pacasmayo, on road to Cajamarca, 19 Mar 1988, *SA Renvoize & S Laegaard 4793* (IBGE 61352, K 3913560, SP 378383).

***Imperata tenuis* Hack.**

**Specimens examined.** BRAZIL. **Goiás:** Santa Rita do Araguaia, on Rio Araguaia, 15 Apr 1930, *A Chase 12033* (RB 51902, US 1448675). **Mato Grosso:** Garimpo de Poxoréu, 08 Aug 1973, *L Arzivenco 602* (ICN 034440). Poconé, Fazenda do Macaco, rodovia 112, aproximadamente no km 17, 07 Jun 1992, *M Schessl 107.4* (CEN 49565). **Mato Grosso do Sul:** Corumbá, Fazenda Santa Filomena, 21 Jun 1979, *GT Prance 26283* (CEN 3390). Corumbá, início do aterro da rodovia Transpantaneira, logo ao sair da cidade, 09 Oct 1985, *JFM Valls 9057* (CEN 107467). Corumbá, 6,6 km ao sul da Curva Leque e 14,6 km do rio Abobral, na estrada do Porto da Manga ao Morro do Azeite, 27 Oct 1986, *JFM Valls 10353* (CEN 115523). **Minas Gerais:** Buritizeiro, Córrego Jenipapo, ao longo da rodovia BR-365, cerca de 10 km a sudeste de Pirapora, 28 May 1992, *JFM Valls 13072* (CEN 116213). Caxambu, 25 km a oeste na rodovia 267 em direção a Cambuquira, 25 Feb 1976, *G Davidse & WG D’Arcy 10611* (SP 159469). Januária, *WR Anderson 9321* (MBM 72490). Leopoldina, 01 Sep 1988, *MC Brügger 22662* (ICN 133622). Parque Nacional Grande Sertão Veredas, 02 May 1999, *RR Silva et al. 386* (SP 335614). São João del-Rei, 1888, *AFM Glaziou 17442* (K 632952, K 632953, P 740687, P 740688, P 740689, US 2942435, W 1916-0034901). São João Del-Rei, Bengo, 30 May 1989, *L Krieger & MC Brügger 23776* (ICN 133394). São Sebastião da Vitória, 16 May 1988, *L Krieger 25605* (ICN 133605). Uberlândia, estrada para Campo Florido, 16 Apr 1999, *GM Araújo 2392* (HUFU 19688). Uberlândia, Estação Ecológica do Panga, 21 May 1999, *AAA Barbosa s.n.* (HUFU 20113, HUFU 20114). Uberlândia, estrada para Campo Florido, a 20 km do centro da cidade, 03 Mar 2000, *GM Araújo & S Mendes s.n.* (HUFU 22568). Uberlândia, Estação Ecológica do Panga, 26 May 2000, *AAA Barbosa 3349* (HUFU 23176). Uberlândia, estrada para Campo Florido, a 20 km do centro da cidade, 02 Jun 2000, *S Mendes & AM Guimarães* *s.n.* (HUFU 23514). Uberlândia, Fazenda Rio das Pedras, a 300 m da BR-497, na altura do km 5, 28 Feb 2002, *GC Oliveira 341* (HUFU 42171). Uberlândia, Fazenda Rio das Pedras, a 300 m da BR-497, na altura do km 5, 23 Mar 2002, *GC Oliveira 402* (HUFU 42172). Uberlândia, Fazenda Rio das Pedras, a 300 m da BR-497, 31 May 2002, *GC Oliveira 761* (HUFU 42173). Uberlândia, Reserva Ecológica do Panga, 26 Jun 2023, *BLS Pinto & CAD Welker 06* (HUFU 84279). **Paraná:** rodovia 373, a 11 km de Guarapuava, 10 Dec 1992, *Z Rúgolo et al. 1655* (ICN 101133). **Rio Grande do Sul:** Dom Pedrito, próximo ao km 277 da BR-293, entre Dom Pedrito e Livramento, 17 Nov 2003, *JFM Valls* (CEN 51188). Rosário do Sul, Serra do Caverá, 29 Nov 2016, *PJS Silva-Filho 2294* (ICN 193846). São Borja, BR-285, próximo ao km 654, 30 Mar 2012, *CAD Welker 494* (ICN 182114). São Gabriel, BR-290, próximo ao km 451, 15 Dec 2009, *CAD Welker 219* (HUCS 37373, ICN 168829, SMDB 13151). São Leopoldo, Quinta São Manoel, 15 Nov 1903, *Arechavaleta s.n.* (ICN 014638). **Santa Catarina:** Curitibanos, caminho de Curitibanos-Leblon Régis, a 39 km de Curitibanos, 09 Dec 1992, *Z Rúgolo et al. 1588* (ICN 101066). Florianópolis, Ilha de Santa Catarina, Parque Municipal das Dunas da Lagoa da Conceição, 22 Aug 2004, *TB Guimarães & DB Falkenberg 612* (FLOR 35453). Florianópolis, Ilha de Santa Catarina, Pântano do Sul, 23 Nov 2009, *B Toncic & A Zanin 286* (FLOR 37469). Rodeio, 25 Aug 2012, *LA Funez 835* (FLOR 52695). **São Paulo:** Juquitiba, rodovia BR-116 para Curitiba, 08 Mar 1976, *G Davidse et al. 10920* (MBM 71573). Votuporanga, Bairro Viradouro, 28 Jan 1997, *AD Faria et al. 97/278* (ICN 131595).
